# Risk Factors for Sustained Cholera Transmission, Juba County, South Sudan, 2014

**DOI:** 10.3201/eid2110.142051

**Published:** 2015-10

**Authors:** Thomas T.A. Ujjiga, Joseph F. Wamala, Juma J.H. Mogga, Thabo O. Othwonh, David Mutonga, Asta Kone-Coulibaly, Masood Ali, Allan M. Mpairwe, Abubaker Abdinasir, Mohamed A. Abdi, Zabulon Yoti, Olu Olushayo, Pinyi Nyimol, Riek Lul, Richard L. Lako, John Rumunu

**Affiliations:** Ministry of Health, Juba, South Sudan (T.T.A. Ujjiga, J.J.H. Mogga, T.O. Othwonh, P. Nyimol, R. Lul, R.L. Lako, J. Rumunu);; World Health Organization, Juba (J.F. Wamala, D. Mutonga, A. Kone-Coulibaly, M.A. Shaikh, A.M. Mpairwe, A. Abdinasir, M.A. Abdi, Z. Yoti, O. Olushayo)

**Keywords:** cholera, risk factors, matched case-control study, zoonoses, South Sudan, transmission, sub-Saharan Africa, epidemic, water sanitation, environmental factors, vaccine, diarrheal disease, enteric infections, *Vibrio cholerae*

## Abstract

We conducted a case–control study to identify risk factors for the 2014 cholera outbreak in Juba County, South Sudan. Illness was associated with traveling or eating away from home; treating drinking water and receiving oral cholera vaccination were protective. Oral cholera vaccination should be used to complement cholera prevention efforts.

Cholera is an acute diarrheal disease caused by ingestion of food or water contaminated by the bacteria *Vibrio cholerae*, of which O1 is the most common serogroup in Africa (*1*,*2*). Although the proportion of global cholera cases reported from sub-Saharan Africa decreased from 93%–98% during 2001–2009 to 44% in 2013, cholera remains a major cause of disease epidemics in countries like South Sudan (*3*). In recent years, 4 major cholera outbreaks have occurred in South Sudan: in 2006, the number of cholera cases totaled 19,277 (case-fatality rate [CFR] 2.9%); in 2007, cases totaled 22,412 (CFR 1.8%); in 2008, cases totaled 27,017 (CFR 0.57%); and in 2009, cases totaled 48,035 (CFR 0.13%) (*4**–**6*; Ministry of Health, South Sudan, unpub. data). A previous case–control study conducted during the 2007 cholera outbreak in Juba County, South Sudan, showed that cholera was associated with being a visitor to Juba, having a water source close to the residence, and treating drinking water (because of inadequate treatment techniques); eating hot food was the only factor significantly associated with a lower risk of cholera (*7*).

On May 6, 2015, a cholera outbreak was confirmed in Juba County, South Sudan, during a major humanitarian crisis precipitated by political and ethnic tensions that deteriorated dramatically starting in December 2013. Epidemiologic investigations revealed that the outbreak started on April 23, 2014. We conducted a matched case–control study to identify risk factors for, and protective factors against, illness during the 2014 cholera outbreak in Juba County.

## The Study

Cholera case-patients were identified from updated lists from Juba County’s 5 cholera treatment centers (CTCs), 2 of which were located in camps for internally displaced persons (IDPs; i.e., persons who have left their homes but stayed within their country’s borders). Preventive oral cholera vaccination was conducted in the 2 IDP camps before the outbreak began in Juba. A case-patient was defined as a Juba County resident >2 years of age who 1) had an acute illness characterized by >3 loose, watery stools within 24 hours or 2) was confirmed to be positive for *V. cholerae* infection by rapid diagnostic testing or culture during the cholera outbreak that began in Juba County on April 23, 2014.

For cholera case-patients enrolled in the study, a control matched by neighborhood, sex, and age was identified and invited to participate in the study. The study team traveled to the case-patient’s village and worked with the local village leader or a social mobilization volunteer to identify a matching control from a household within a 100-m radius of the case-patient. A control was a Juba County resident >2 years of age with no history of clinical illness or no laboratory evidence of *V. cholerae* infection during this cholera outbreak. To match case-patients and controls by age, age groups of 3–5 years (e.g., 2–4, 5–9, and 65–69 years of age) were used.

A team of 19 trained research assistants administered a pretested, semistructured questionnaire and conducted environmental assessments to evaluate the use of safe drinking water, improved sanitation facilities, personal and food hygiene, and oral cholera vaccination. Using Epi Info (Centers for Disease Control and Prevention, Atlanta, GA, USA), we calculated matched unadjusted and adjusted odds ratios by using bivariate and multivariate models, respectively, to identify risk factors for cholera. The study was approved by the Ministry of Health’s ethics committee.

A total of 134 matched pairs of case-patients and controls were enrolled in the study during June 26–July 29 in 2014 (Figure). Of the 134 case-patients enrolled, 9 were confirmed by culture and 104 by a cholera rapid diagnostic test (OnSite Rapid Test; CTK Biotech, San Diego, CA, USA); the remaining 21 were identified by epidemiologic linkage (i.e., a resident of Juba >2 years of age with >3 loose stools in 24 hours after the beginning of the cholera outbreak). Eleven cholera case-patients with no matching controls were excluded. Mean delay between admission to the CTC and interview after enrollment in the study was 21 days (range 0–55 days); most case-patients who were interviewed were admitted during the peak transmission phase of the outbreak (Figure). The distribution of age, sex, residence of origin, education level, and occupation were comparable among case-patients and controls (Table 1). Most (118 [88%]) case-patients enrolled in the study visited the CTC within 1 day of onset of cholera symptoms. All 134 patients had diarrhea; 112 (84%) had vomiting; 45 (34%) had abdominal cramps; 37 (28%) had some dehydration (i.e., any 2 signs of dehydration, including 1 major sign); and 31 (23%) had severe dehydration (23%).

**Figure:**
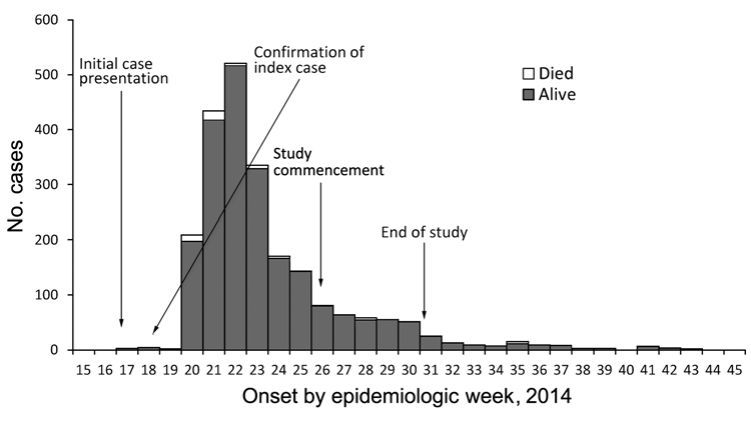
Timeline showing number of cholera cases (total cases = 2,260), deaths from cholera (total deaths = 43; case fatality rate 2.0%), and dates of study for cholera outbreak in Juba County, South Sudan, epidemiologic weeks 17–43 (April 23–October 20), 2014.

**Table 1:** Characteristics of cholera case-patients and controls during outbreak in Juba County, South Sudan, 2014

Characteristic	Case-patients, no. (%), N = 134	Controls, no. (%), N = 134	p value
Age group, y			0.99
0–9	59 (45)	60 (45.5)	
10–19	4 (3)	5 (3.8)	
20–29	28 (21.2)	27 (20.6)	
30–39	20 (15.2)	21 (16)	
40–49	14 (10.6)	14 (10.7)	
50–59	5 (3.8)	4 (3.1)	
>60	1 (0.8)	1 (0.8)	
Sex			1.0
M	67 (50)	67 (50)	
F	67 (50)	67 (50)	
Payam of origin*			0.99
Juba	47 (35.0)	47 (35.0)	
Northern Bari	36 (27.0)	36 (27.0)	
Rejaf	30 (22.4)	30 (22.4)	
Munuki	6 (4.5)	6 (4.5)	
Kator	7 (5.2)	7 (5.2)	
Others	8 (5.9)	8 (5.9)	
Education level			0.85
None	87 (65.4)	88 (65.7)	
Primary/tertiary	46 (34.6)	46 (34.3)	
Employment status			0.18
Unemployed	100 (81.3)	112 (86.2)	
Employed	23 (18.7)	18 (13.8)	

*Payams are administrative divisions (counties) in South Sudan.

Bivariate and multivariate analyses showed that persons who ate food outside their home before illness onset and those who traveled outside their home village (even within the county) before illness onset were significantly more likely to develop cholera (Table 2). Conversely, treating drinking water at home and receiving >2 doses of oral cholera vaccine (self-reported) were protective against cholera (Table 2). Eating outside the home as a risk factor in this cholera outbreak is consistent with findings from cholera outbreaks in Uganda and Haiti (*8**,**9*). Popular eating places in Juba County included roadside food vendors and restaurants in markets that did not meet minimum food hygiene standards yet remained open during the outbreak because public health inspection of eating establishments and a ban on roadside food vending were not uniformly enforced. Our study identified recent travel to cholera outbreak areas as a risk factor, also a finding consistently associated with cholera spread to new locations during previous cholera outbreaks (*10*).

**Table 2:** Factors examined by using bivariate and multivariate analyses during cholera outbreak in Juba County, South Sudan, 2014*

Factor	Case-patients, no. (%), N = 134	Controls, no. (%), N = 134	Unadjusted matched			Adjusted matched	
			OR (95% CI)	p value		OR (95% CI)	p value
Ate outside home before illness							
Yes	42 (31.6)	17 (14.0)	6.5 (2.27–18.62)	<0.001		9.17 (1.89–44.41)	0.006
No	91 (68.4)	104 (86.0)					
Traveled outside home village before onset of illness†							
Yes	37 (28.5)	10 (7.9)	13 (3.09–54.77)	<0.0001		10.14 (1.75–58.87)	0.01
No	93 (71.5)	117 (92.1)					
Treated drinking water at home							
Yes	51 (38.3)	58 (44.3)	0.11 (0.02–0.55)	0.04		0.10 (0.02–0.72)	0.02
No	82 (61.7)	73 (55.7)					
Had 2 oral cholera vaccine doses‡							
Yes	55 (41.7)	78 (59.5)	0.08 (0.02–0.35)	<0.001		0.10 (0.02–0.65)	0.016
No	77 (58.3)	53 (40.5)					

*The first 2 factors increased risk for cholera, whereas the other 2 factors decreased risk. OR, odds ratio. †Travel from home to any area affected by cholera during the 2014 outbreak in South Sudan. ‡Self-reported.

Also, as reported in previous cholera outbreaks in South Sudan, Uganda, Haiti, and Zimbabwe, household chlorination of drinking water was associated with significantly lower risk for developing cholera in our study (*7**,**8**,**9**,**11*). In our study, water samples from case-patient households that did not chlorinate their drinking water showed evidence of contamination with fecal coliforms (>10 counts/100 mL). Similarly, water samples from water storage vessels in 2 case-patient households that did not treat their drinking water were contaminated with fecal coliforms (>10 counts/100 mL).

Oral cholera vaccination is known to confer protection from cholera (*12*). We found that oral cholera vaccination was associated with a significantly reduced risk of cholera infection and a vaccine effectiveness of 90% (Table 2).

Our findings are subject to several limitations that could potentially have confounded our results. These limitations include underreporting of high-risk behaviors, recall bias, potential misclassification of asymptomatic case-patients, narrow age ranges that caused difficulty in identifying matching controls, shared environmental risk factors (e.g., shared water source) for case-patients and controls, unmeasured variables (i.e., factors not measured in this study, such as being an IDP), and loss of oral cholera vaccination cards (i.e., vaccinations were self reported).

## Conclusions

For this cholera outbreak in South Sudan, we found that travel and eating outside the home were risk factors for becoming ill and that treating drinking water at home and getting oral cholera vaccination provided protection against illness. For cholera prevention and control in humanitarian crises, we recommend that global oral cholera vaccine stockpiles be enhanced so that preventive oral cholera vaccination can be used to augment traditional interventions, such as improved access to safe drinking water and public education about risk factors.
